# Lymphopenia after palliative radiotherapy for vertebral metastases

**DOI:** 10.1093/jrr/rrae038

**Published:** 2024-05-31

**Authors:** Kazuya Takeda, Rei Umezawa, Takaya Yamamoto, Noriyoshi Takahashi, Yu Suzuki, Keita Kishida, So Omata, Keiichi Jingu

**Affiliations:** Department of Radiation Oncology, Tohoku University Graduate School of Medicine, 1-1 Seiryo-machi, Aoba-ku, Sendai 980-8574, Miyagi Japan; Department of Radiation Oncology, South Miyagi Medical Center, 38-1 Nishi, Ogawara, Shibata 989-1253, Miyagi, Japan; Department of Radiation Oncology, Tohoku University Graduate School of Medicine, 1-1 Seiryo-machi, Aoba-ku, Sendai 980-8574, Miyagi Japan; Department of Radiation Oncology, Tohoku University Graduate School of Medicine, 1-1 Seiryo-machi, Aoba-ku, Sendai 980-8574, Miyagi Japan; Department of Radiation Oncology, Tohoku University Graduate School of Medicine, 1-1 Seiryo-machi, Aoba-ku, Sendai 980-8574, Miyagi Japan; Department of Radiation Oncology, Tohoku University Graduate School of Medicine, 1-1 Seiryo-machi, Aoba-ku, Sendai 980-8574, Miyagi Japan; Department of Radiation Oncology, Tohoku University Graduate School of Medicine, 1-1 Seiryo-machi, Aoba-ku, Sendai 980-8574, Miyagi Japan; Department of Radiation Oncology, Tohoku University Graduate School of Medicine, 1-1 Seiryo-machi, Aoba-ku, Sendai 980-8574, Miyagi Japan; Department of Radiation Oncology, Tohoku University Graduate School of Medicine, 1-1 Seiryo-machi, Aoba-ku, Sendai 980-8574, Miyagi Japan

**Keywords:** lymphopenia, lymphocytopenia, palliative radiotherapy, vertebral metastasis

## Abstract

Lymphopenia is a well-known side effect of radiotherapy and has been shown to have a negative impact on patient outcomes. However, the extent of lymphopenia caused by palliative radiotherapy and its effect on patient prognosis has not been clarified. The aim of this study was to determine the incidence and severity of lymphopenia after palliative radiotherapy for vertebral metastases and to determine their effects on patients’ survival outcomes. We conducted a retrospective analysis for patients who underwent palliative radiotherapy for vertebral metastases and could be followed up for 12 weeks. Lymphocyte counts were documented at baseline and throughout the 12-week period following the start of radiotherapy and their medians and interquartile ranges (IQRs) were recorded. Exploratory analyses were performed to identify predictive factors for lymphopenia and its impact on overall survival (OS). A total of 282 cases that met the inclusion criteria were analyzed. The median baseline lymphocyte count was 1.26 × 10^3^/μl (IQR: 0.89–1.72 × 10^3^/μl). Peak lymphopenia occurred at a median of 26 days (IQR: 15–45 days) with a median nadir of 0.52 × 10^3^/μl (IQR: 0.31–0.81 × 10^3^/μl). Long-term analysis of patients surviving for 1 year showed that lymphopenia persisted at 1 year after radiotherapy. The main irradiation site, radiation field length and pretreatment lymphocyte count were significantly related to grade 3 or higher lymphopenia. Lymphopenia was identified as a significant predictor of OS by multivariate Cox regression analysis. This study demonstrated the incidence of lymphopenia after palliative radiotherapy for vertebral metastases and its effect on patients’ OS.

## INTRODUCTION

Lymphocytes are highly sensitive to radiation, which makes them vulnerable to interphase death even with exposure to low doses of radiation [1]. In cancer treatment, radiation therapy has been known to cause lymphopenia [2, 3]. We have previously reported that the overall incidence rate of grade 3 lymphopenia following radiotherapy was 45% [3]. In that study, lymphopenia was observed across various types of radiotherapy, including different irradiation sites, field sizes and dose fraction schedules. The negative impact of lymphopenia on patient treatment outcomes and overall survival (OS) has recently been reported and has attracted increased attention [4, 5]. Palliative radiotherapy is commonly used in cancer treatment for vertebral metastases due to its minimal side effects. However, the details of lymphopenia induced by palliative radiotherapy, including its risk factors and impact on prognosis, still need to be explored.

This study was conducted to investigate the details of lymphopenia following palliative radiotherapy for vertebral metastases, including its incidence, risk factors and effects on patient outcomes.

## MATERIALS AND METHODS

### Study design

This study received approval from the Institutional Review Board of Tohoku University Graduate School of Medicine (2021-1-1073). This study was a sub-analysis of a previous study in which prognostic factors for patients with vertebral metastases were investigated [6], and it partially overlaps with another study in which blood cell reduction after radiation monotherapy was comprehensively analyzed [3]. The methodology is described only briefly here as the previous publications contain detailed information about the patient cohort and treatment methods. The study included patients who received palliative radiotherapy for vertebral metastases at a single institution from 2010 to 2020. In the original study, 487 patients were identified after excluding those with (i) primary vertebral bone malignancies, (ii) stereotactic body radiotherapy, (iii) concurrent irradiation for lesions other than those in vertebral bone, (iv) a history of vertebral bone radiotherapy or radiotherapy to any site within 12 weeks of the current treatment course or (v) missing blood test data for more than half of the parameters. From this cohort, 282 cases that met the following criteria were used for analysis in this study: (i) a single treatment course (multiple site irradiations in a single treatment course being allowed), (ii) OS of more than 12 weeks from the start of radiotherapy and (iii) at least one lymphocyte count examination within 12 weeks of the start of radiotherapy.

### Data interpretation

Clinical and hematological data were obtained from institutional clinical records. The general condition for each patient was assessed by using the ECOG Performance Status Scale. The sites of the primary disease were classified into a low-risk group (prostate cancer, breast cancer and hematological malignancy) and a high-risk group (other malignancies) based on previous reports [6–8]. Analgesia use was determined from the prescription record in a 12-week period before the start of radiotherapy. Radiation history was obtained from the hospital radiation information system. For chemotherapy use, the prescription of all oral and injectable chemotherapy drug, excluding hormonal therapy drugs, was recorded. To assess their impact on lymphopenia, chemotherapy use within a 12-week period before the start of radiotherapy was defined as the chemotherapy record, and chemotherapy use within a 12-week period after the start of radiotherapy was defined as concurrent chemotherapy. For corticosteroid use, the prescription of all oral and injectable steroids was recorded. As with chemotherapy use, history and concurrent use of corticosteroids was defined as use in the 12-week period before and after the start of radiotherapy. The length of the radiation field was defined as the maximum jaw opening in all treatment fields. In patients who received multiple site irradiation, the sum of the field lengths of all treatment plans was recorded. As a surrogate marker of infectious disease, the use of antibiotics within a 12-week period after the end of radiotherapy was recorded. OS was estimated from the start of radiotherapy until the occurrence of death or the last follow-up.

Lymphocyte counts were measured at baseline (pretreatment) and throughout the 12-week period following the start of radiotherapy. The lowest lymphocyte count and the timing of its occurrence were documented for each patient. To analyze changes over time, the most recent lymphocyte count was considered to be sustained until the next blood test. The severity of lymphopenia was assessed using the Common Terminology Criteria for Adverse Events (CTCAE) Version 5.0. As part of a long-term exploratory analysis, we identified patients who survived for 1 year or longer and had undergone blood tests four or more times during that period. Among those patients, the time-course change of median lymphocyte counts was plotted over a period of 1 year after radiotherapy.

### Statistical analysis

For the baseline analysis, each variable was presented as a number with percentage or as a median with interquartile range (IQR), as appropriate. A paired *t*-test was used to estimate the change in lymphocyte count, and Bonferroni correction was applied to correct for multiple comparisons as appropriate. For visualization purposes, the weekly median lymphocyte count and its IQR were plotted. The logistic regression model was used to identify predictive factors for grade 3 lymphopenia. The Cox proportional hazards regression model was used to examine the effect of lymphopenia and other clinical factors on patient survival outcomes. In the regression analyses, factors with a *P*-value of less than 0.1 in the univariate analysis were included in the multivariate analysis. The correlation between lymphopenia and antibiotic use was evaluated using the chi-squared test. Survival curves were plotted using the Kaplan–Meier method, and the log-rank test was used to evaluate the difference between survival curves. Statistical significance was determined by a *P*-value of less than 0.05 for each test. All statistical analyses were performed with JMP Pro 17.1.0 (JMP Statistical Discovery LLC, NC) and R 4.2.2 (The R Foundation for Statistical Computing Platform, Vienna, Austria).

## RESULTS

**Fig. 1:**
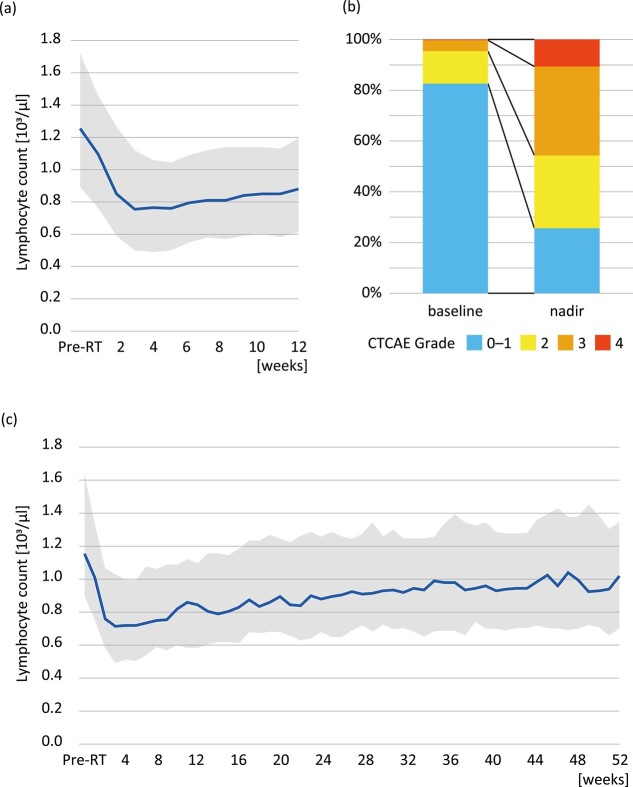
Lymphocyte counts before and after radiotherapy. (a) Time-course changes of lymphocyte count shown as median and IQR over a 12-week period for all cases, (b) CTCAE grades before the start of radiotherapy and at nadir and (c) long-term analysis in patients who survived for 1 year or longer.

A total of 282 cases met the eligibility criteria, and patient characteristics are summarized in Table 1. The median follow-up of was 14.8 months for the entire cohort and 22.7 months for those who were alive. Figure 1a shows the time course of the median lymphocyte count. The median lymphocyte count before radiation therapy was 1.26 × 10^3^/μl (IQR: 0.89–1.72 × 10^3^/μl) and the median nadir was 0.52 × 10^3^/μl (IQR: 0.31–0.81 × 10^3^/μl, *P* < 0.001 compared with baseline). Figure 1b shows the proportions of patients with lymphopenia before the start of radiotherapy and at nadir after the start of radiotherapy. The CTCAE grades of lymphopenia at nadir were as follows: grade 0–1 in 72 cases (26%), grade 2 in 81 cases (29%), grade 3 in 99 cases (35%) and grade 4 in 30 cases (11%). The median duration from the beginning of radiotherapy to nadir was 26 days (IQR: 15–45 days). Figure 1c shows the median lymphocyte count over a period of 1 year in the 110 patients who survived for more than 1 year of survival. The median lymphocyte count before radiation therapy was 1.16 × 10^3^/μl (IQR: 0.90–1.64 × 10^3^/μl), the median nadir was 0.50 × 10^3^/μl (IQR: 0.31–0.70 × 10^3^/μl, *P* < 0.001 compared with baseline) and the median lymphocyte count at 1 year after the start of radiotherapy was 1.02 × 10^3^/μl (IQR: 0.70–1.35 × 10^3^/μl, *P* = 0.002 compared with baseline). Lymphocyte counts had not returned to baseline counts 1 year after therapy despite the gradual recovery of lymphocyte counts over time.

**Table 1:** Patient characteristics. Values are presented as number with percentage or median with IQR

**Characteristic**	***n* = 282**
Age	65 (57, 72)
Sex	
Female	132 (47%)
Male	150 (53%)
Performance status	
0–1	62 (22%)
2	56 (20%)
3	18 (6.4%)
4	14 (5.0%)
Unknown	132 (47%)
Primary disease	
Head and neck	17 (6.0%)
Lung, mediastinum	54 (19%)
Breast	48 (17%)
Gastrointestinal tract	38 (13%)
Liver, bile duct, pancreas	19 (6.7%)
Urinary tract	24 (8.5%)
Gynecologic organ	10 (3.5%)
Prostate	25 (8.9%)
Hematological malignancy	21 (7.4%)
Bone, skin, soft tissue	18 (6.4%)
Other, unknown	8 (2.8%)
Risk of primary disease	
Low risk	94 (33%)
High risk	188 (67%)
Analgesia use	
No	47 (17%)
Yes	235 (83%)
Radiotherapy history	
No	211 (75%)
Yes	71 (25%)
Chemotherapy history	
No	128 (45%)
Yes	154 (55%)
Concurrent chemotherapy	
No	132 (47%)
Yes	150 (53%)
Glucocorticoid use history	
No	189 (67%)
Yes	93 (33%)
Concurrent glucocorticoid use	
No	156 (55%)
Yes	126 (45%)
Main irradiation site	
Cervical	34 (12%)
Lumbar	96 (34%)
Thoracic	119 (42%)
Multiple sites	33 (12%)
Number of irradiation sites	
1	249 (88%)
2	32 (11%)
3	1 (0.4%)
Biological effective dose (α/β = 10)	39 (39, 47)
Dose fractionation	
1–9	36 (13%)
10	144 (51%)
11–	102 (36%)
Overall treatment time	15 (14, 22)
Total length of radiation field [cm]	10.9 (8.7, 16.1)
Pretreatment lymphocyte count	1.26 (0.89, 1.72)
Pretreatment lymphopenia grade	
Grade 0–1	233 (83%)
Grade 2	36 (13%)
Grade 3	12 (4.3%)
Grade 4	1 (0.4%)

Chemotherapy is categorized as history (up to the day before the start of irradiation) and concurrent (used within 12 weeks of the start of radiotherapy). Primary tumors other than low-risk tumors (prostate, breast and hematologic neoplasms) were defined as a high-risk group.

Table 2 shows the results of logistic regression analysis of predictive factors for developing grade 3 or higher lymphopenia. Multivariate analysis showed that irradiation to the thoracic vertebrae, length of the irradiation field, concurrent glucocorticoid use and pretreatment lymphocyte count were statistically significant factors related to grade 3 or higher lymphopenia.

**Table 2:** Analysis of predictive factors for grade 3 or higher lymphopenia

**Characteristic**	**Univariate analysis**			**Multivariable analysis**		
	**Hazzard ratio**	**95% Confidence Interval**	***P*-value**	**Hazzard ratio**	**95% Confidence Interval**	***P*-value**
Age	1.00	0.98, 1.02	0.8			
Sex			0.5			
Male	—	—				
Female	1.16	0.73, 1.86				
Performance status			0.1			
0–2, Unknown	—	—				
3–4	1.86	0.89, 4.01				
Analgesia use			**0.005**			0.2
No	—	—		—	—	
Yes	2.55	1.31, 5.24		1.74	0.75, 4.21	
Radiotherapy history			0.07			0.4
No	—	—		—	—	
Yes	1.64	0.96, 2.83		1.34	0.67, 2.72	
Risk of primary disease			0.8			
High risk	—	—				
Low risk	0.94	0.57, 1.54				
Main irradiation site			**0.002**			**0.02**
Thoracic	—	—		—	—	
Cervical	0.26	0.10, 0.59		0.26	0.09, 0.67	
Lumbar	0.52	0.30, 0.90		0.50	0.25, 0.97	
Multiple sites	1.13	0.52, 2.49		0.68	0.21, 2.13	
Chemotherapy history			0.4			
No	—	—				
Yes	0.82	0.51, 1.31				
Concurrent chemotherapy			0.08			>0.9
No	—	—		—	—	
Yes	1.53	0.96, 2.46		1.01	0.52, 1.94	
Glucocorticoid use history			0.4			
No	—	—				
Yes	1.25	0.76, 2.06				
Concurrent glucocorticoid use			**<0.001**			**0.04**
No	—	—		—	—	
Yes	2.61	1.62, 4.26		1.94	1.02, 3.73	
Biological effective dose (α/β = 10)	0.99	0.97, 1.01	0.4			
Dose fractionation			**0.047**			0.2
10	—	—		—	—	
1–9	0.82	0.39, 1.71		0.53	0.21, 1.30	
11–	0.52	0.31, 0.88		0.65	0.34, 1.24	
Overall treatment time	0.99	0.97, 1.02	0.5			
Total length of radiation field	1.07	1.03, 1.12	**0.001**	1.10	1.03, 1.17	**0.002**
Irradiation for multiple sites			0.15			
No	—	—				
Yes	1.71	0.83, 3.63				
Pretreatment lymphocyte count (per 100/μl)	0.83	0.78, 0.87	**<0.001**	0.83	0.77, 0.88	**<0.001**

Primary tumors other than low-risk tumors (prostate, breast and hematologic neoplasms) were defined as a high-risk group.

As a surrogate indicator of infection, the use of antibiotics within a 12-week period after completion of radiotherapy was investigated. Prescription of antibiotics was observed in 19% of the patients with grade 3–4 lymphopenia and 9% of the patients with grade 0–2 lymphopenia (*P* = 0.02).

**Fig. 2:**
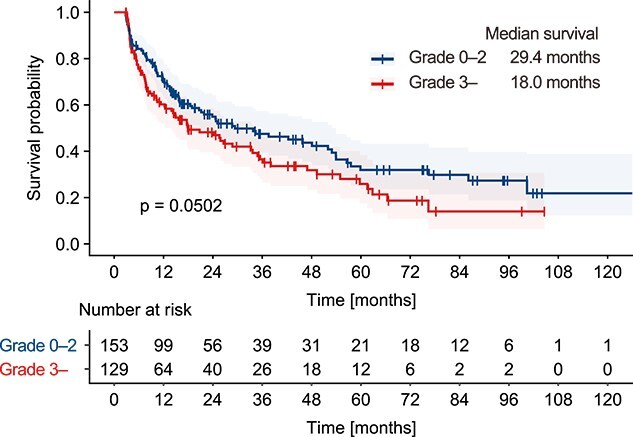
Survival analysis. Patients were stratified by the occurrence of grade 3 lymphopenia. The survival curves were plotted using the Kaplan–Meier method and differences between groups were evaluated with the log-rank test.

In the survival analysis, the median OS of all patients was 25.1 months. A lower lymphocyte nadir after radiotherapy was identified by Cox regression model analysis as a statistically significant predictor of shorter OS (Table 3). Other factors including performance status, analgesia use, type of primary disease, history of chemotherapy use, concurrent chemotherapy, concurrent corticosteroid use and dose fractionation of radiotherapy were also significant predictors. As for anticancer drugs, the history of chemotherapy was associated with shorter OS, while concurrent chemotherapy use was associated with longer OS. Figure 2 shows survival curves stratified by the incidence of grade 3 or higher lymphopenia. The median OS periods for patients with and those without grade 3 or higher lymphopenia were 18.0 and 29.4 months, respectively. Although not statistically significant, patients with grade 3 or higher lymphopenia tended to have shorter OS based on the log-rank test (*P* = 0.05, Fig. 2).

**Table 3:** Cox hazard analysis for patient OS

		**Univariate analysis**			**Multivariate analysis**		
**Characteristic**	**Category**	**Hazzard ratio**	**95% Confidence Interval**	***P*-value**	**Hazzard ratio**	**95% Confidence Interval**	***P*-value**
Age		1.01	1.00, 1.02	0.2			
Sex	Female vs Male	0.76	0.56, 1.04	0.09	0.78	0.56, 1.08	0.13
Performance status	3–4 vs 0–2, Unknown	1.52	0.95, 2.44	0.08	1.78	1.04, 3.05	**0.04**
Analgesia use	Yes vs No	2.69	1.60, 4.51	**<0.001**	1.88	1.07, 3.30	**0.03**
Radiotherapy history	Yes vs No	1.37	0.97, 1.95	0.08	1.44	0.95, 2.17	0.08
Risk of primary disease	Low risk vs High risk	0.35	0.24, 0.51	**<0.001**	0.43	0.28, 0.66	**<0.001**
Main irradiation site	Thoracic	—	—				
	Cervical	0.91	0.55, 1.51	0.7			
	Lumbar	1.07	0.75, 1.52	0.7			
	Multiple sites	0.88	0.51, 1.52	0.6			
Chemotherapy history	Yes vs No	2.41	1.74, 3.35	**<0.001**	2.08	1.40, 3.10	**<0.001**
Concurrent chemotherapy	Yes vs No	1.71	1.24, 2.35	**0.001**	0.57	0.38, 0.87	**0.01**
Corticosteroid use history	Yes vs No	2.16	1.57, 2.97	**<0.001**	1.36	0.91, 2.05	0.1
Concurrent corticosteroid use	Yes vs No	2.26	1.64, 3.10	**<0.001**	1.62	1.06, 2.49	**0.03**
Biological effective dose (α/β = 10)		0.96	0.94, 0.97	**<0.001**	0.99	0.95, 1.03	0.5
Dose fractionation	10	—	—		—	—	
	1–9	1.12	0.71, 1.77	0.6	1.01	0.53, 1.93	>0.9
	11–	0.31	0.21, 0.45	**<0.001**	0.47	0.25, 0.88	**0.02**
Overall treatment time		0.95	0.93, 0.97	**<0.001**	1.00	0.96, 1.05	0.9
Total length of radiation field		1.01	0.99, 1.04	0.3			
Pretreatment lymphocyte count (per 100/μl)		0.97	0.94, 0.99	**0.02**	1.03	1.00, 1.06	0.08
Nadir lymphocyte count (per 100/μl)		0.93	0.89, 0.98	**0.002**	0.94	0.88, 0.99	**0.02**

## DISCUSSION

Lymphopenia after palliative radiotherapy for vertebral metastases was investigated in this study. To the best of our knowledge, this is the first in-depth study on lymphopenia in these patients. Lymphocytes are highly sensitive to radiation and are known to undergo interphase death after radiotherapy [1]. Additionally, there is evidence suggesting that radiation-induced increase in myeloid-derived suppressor cells may contribute to lymphopenia after radiotherapy [9]. Previous studies have established a clinical correlation between lymphopenia and radiotherapy in patients with brain, head and neck, lung, esophageal, breast and cervical cancer [10–15]. In our recent study, we observed a reduction in blood cell count in a cohort of patients who received radiation monotherapy for various diseases [3]. In that study, Grade 3–4 lymphocytopenia after radiotherapy was observed in 45%. Notably, lymphopenia was observed even in short-term palliative radiotherapy for sites such as the vertebrae, which had received less attention previously. Therefore, the occurrence of lymphopenia and its predictive factors in patients receiving radiotherapy to the vertebrae were analyzed in detail in this study.

In this study, the lymphocyte counts decreased sharply from a median baseline of 1.26 × 10^3^/μl to 0.52 × 10^3^/μl within a median period of 26 days. In the 12-week observation period, 46% of the patients underwent grade 3–4 lymphopenia. Subgroup analysis revealed that the lymphocyte counts had not returned to baseline levels even 1 year after the start of irradiation. This extended recovery period may be due to the fact that some lymphocytes have a lifespan of several years. Logistic regression analysis showed that baseline lymphocyte count, treatment site, length of the irradiation field and concurrent glucocorticoid use were statistically significant predictors of grade 3 or higher lymphopenia. Treatment site and field size have been reported to be related to lymphocytopenia in association with the volume of irradiated circulating lymphocytes [16–19]. Among those studies, our study is the first study to confirm the effects of these factors on the incidence of lymphopenia in patients who underwent palliative radiotherapy for vertebral bone. These findings suggest that limiting irradiation sites and treatment margins may help prevent the incidence of lymphopenia. The use of glucocorticoid or steroid drugs had been reported to be associated with lymphopenia in healthy populations [20]. Changik *et al.* [21] reported a relation between high-dose glucocorticoid use and high-grade lymphopenia in patients with glioblastoma treated with chemoradiotherapy. These findings were consistent with our study. On the other hand, the use of cytotoxic chemotherapy, which was examined in previous studies, was not associated with severe lymphopenia in our study. This may be because the use of chemotherapy is related to relatively reserved general and bone marrow conditions in the study population.

Severe lymphopenia has been identified as a predictive factor for unfavorable outcomes following radiotherapy for various diseases [5, 17, 22, 23]. Grossman *et al* [24]*.* reported the effect of total lymphocyte count on OS in patients with malignant glioma, pancreatic cancer and stage III non-small cell lung cancer. In that study, median OS was 18 months in patients with a lymphocyte count of 500 or more at 2 months after the start of radiotherapy and 15 months in patients with a lymphocyte count less than 500, respectively (hazard ratio: 2.1, 95% confidence interval: 1.54–2.78, *P*-value: <0.0001). In the present study, Cox regression analysis revealed that a lower lymphocyte nadir after radiotherapy was a statistically significant predictor of shorter OS along with other factors including performance status, analgesia use, type of primary disease and history of chemotherapy use.

The mechanism of the association between lymphopenia and poor survival is not fully understood. Since we could not collect data on the cause of death in this study, we investigated the use of antibiotics to assess the risk of infection due to lymphopenia. We found that the rate of antibiotic use was doubled in patients with grade 3–4 lymphopenia compared with patients with grade 0–2 lymphopenia (19% vs 9%, *P* = 0.02). This suggests that lymphopenia might be associated with patients’ poor prognosis via infectious disease. Terrones *et al.* [25, 26] reported that lymphopenia was associated with increased risk of infection, which is consistent with our results. On the other hand, Grossman *et al.* [27] reported that death from infection was observed in only 2.5% of patients in 96 glioblastoma patients treated with chemoradiotherapy, in which 73% of patients experienced a decline in CD4-positive lymphocyte count to less than 300/mm^3^. Another possible explanation for shorter OS is that suppressed anti-tumor immunity contributes to oncologic progression, but there is insufficient evidence in the field of radiation oncology and future study is warranted.

Although there is a limitation in this study, which includes only patients who survived 12 weeks or longer for analysis of lymphocyte counts, the results of this study suggest that lymphopenia after radiotherapy should be noted even in palliative radiotherapy settings. Considering that other predictive factors revealed in this study, including performance status, analgesia use, primary disease site and history of chemotherapy use, were non-modifiable factors in a clinical situation, minimizing radiation fields may be a better treatment strategy. For instance, it may be preferable not to include adjacent vertebrae of the disease site in the clinical target volume if there is no suspicious region. Additionally, reducing treatment margins through patient fixation and the use of image-guided radiotherapy techniques may also help minimize the irradiated volume. Although intensity-modulated radiotherapy and particle therapy have the potential to reduce normal tissue irradiation, their accessibility may be limited in real clinical situations. Recently, advanced models for predicting radiation-induced lymphopenia using blood distribution modeling or voxel-based modeling have been proposed [28]. These approaches could be potentially helpful in selecting patients who would benefit from more advanced irradiation techniques to minimize the irradiated volume.

This study has several limitations due to its retrospective study design and limited availability of data. Firstly, performance status, which is one of the most common indicators of the general condition of patients, was not available in 47% of the study population, as well as there may be uncertainty in evaluating the performance status of each patient. This may limit the certainty in estimating predictive factors for lymphopenia and OS. Secondly, only a small number of cases involving single-fraction irradiation, a method established for pain relief radiotherapy, were included. The potential benefits of single-dose radiation therapy, such as reducing the exposure of circulating lymphocytes and minimizing radiation-induced lymphopenia, were not fully explored. Further research on single-dose radiation therapy is therefore necessary. Thirdly, there could be confounding due to the potentially correlated parameters included in the multivariate analyses. A prospective study or an appropriate analysis of a large, well-controlled cohort is needed. Fourthly, the study did not include dose–volume analysis for the body and various organs to predict lymphopenia, which should be investigated further in future studies.

## CONCLUSION

In conclusion, we have provided insights into the incidence of lymphopenia and its risk factors following palliative radiotherapy for vertebral metastases. We have also revealed the negative impact of lymphopenia on patients’ survival outcomes. These findings may assist clinicians in optimizing their radiotherapy strategies.
